# The use of serum alkaline phosphatase as a choledocholithiasis marker to mitigate the cost of magnetic resonance cholangiography

**DOI:** 10.31744/einstein_journal/2023AO0204

**Published:** 2023-07-31

**Authors:** Pedro Henrique Peixoto Costa, Jorge Henrique Bento de Sousa, Ian Torres de Lima, Marcos Antonio Neves Noronha, Gabriel Lunardi Aranha, Vitor Pelogi Arienzo, Phellipe Fabbrini Santos Lucas, Milton Steinman, Francisco Tustumi

**Affiliations:** 1 Hospital Israelita Albert Einstein São Paulo SP Brazil Hospital Israelita Albert Einstein, São Paulo, SP, Brazil.

**Keywords:** Cholelithiasis, Choledocholithiasis, Gallstones, Alkaline phosphatase, Cholangiopancreatography, magnetic resonance, Health care costs

## Abstract

**Objective:**

To assess the predictive value of preoperative serum laboratory test results for identifying choledocholithiasis and reduce the use of cholangioresonance and its inherent costs.

**Methods:**

Patients aged 21-69 years who underwent preoperative cholangioresonance examination at our institute were included. Patients with a history of fluctuating jaundice or biliary pancreatitis, bile duct dilatation on ultrasonography, and elevated levels of canalicular enzymes (alkaline phosphatase >100U/L and gamma-glutamyl transferase >50U/L) underwent cholangioresonance-guided surgery. Cases of choledocholithiasis confirmed by cholangioresonance were compared with those without choledocholithiasis. Serum laboratory data were evaluated and the diagnostic capabilities of these examinations were analyzed.

**Results:**

A total of 104 patients were included. For detecting choledocholithiasis using alkaline phosphatase, the cut-off point was 78U/L, sensitivity was 97.6% (95%CI: 87.4-99.9), and specificity was 72.6% (95%CI: 59.8-83.1). In the binary logistic regression analysis, age (OR= 0.92; 95%CI: 0.86-0.98) and alkaline phosphatase level (OR= 1.02; 95%CI: 1.01-1.05) were selected for the final model.

**Conclusion:**

Serum alkaline phosphatase levels may aid preoperative diagnosis of asymptomatic choledocholithiasis. After a global clinical assessment of the patient, serum laboratory findings may contribute to a reduction in cholangioresonance-related heathcare costs.

**Figure:**
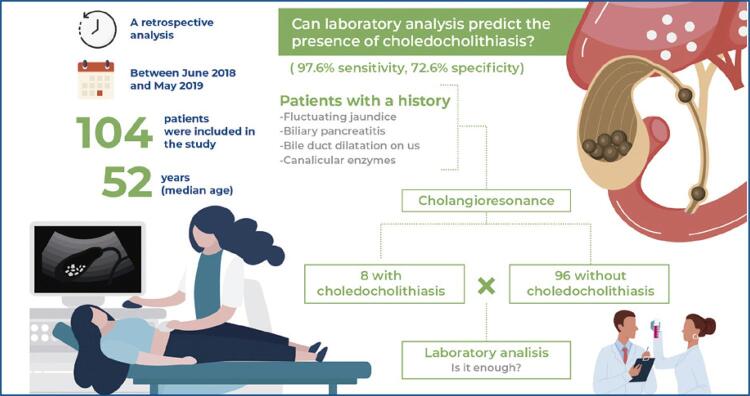

## INTRODUCTION

Choledocholithiasis occurs in approximately 11–21% of patients.^(1)^ Preoperative suspicion and diagnosis are extremely important because of the potential for severe complications. Choledocholithiasis is classified as primary when the stones originate in the common bile duct or as secondary when the stones migrate from the gallbladder, which is more common. The typical clinical presentations are pain and cholestatic syndrome, which may progress to ascending cholangitis and septic shock in extreme cases.^(1)^ However, most patients are asymptomatic.^(1)^

There are several predictive factors for choledocholithiasis, including changes observed in laboratory test results such as increased bilirubin, alkaline phosphatase (ALP), gamma-glutamyl transferase, and hepatic transaminase levels and ultrasound findings such as bile duct dilatation. However, laboratory test results may be vary even in the absence of choledocholithiasis due to nonspecific reactive hepatitis, especially in cases of acute cholecystitis. Furthermore, abdominal ultrasound does not have high accuracy for detecting common bile duct stones (sensitivity, 73%; specificity, 91%).^(2,3)^ For detecting choledocholithiasis, diverse diagnostic tests are available; however, no literary consensus exists regarding their use. This suggests the need for further studies to broaden the understanding of the impact of each of these tests to optimize the investigation for the benefit of the patient and reduce costs of related healthcare services.

Generally, patients are stratified according to the risk of choledocholithiasis into high-, moderate-, and low-risk groups based on the American Society for Gastrointestinal Endoscopy guidelines.^(3,4)^ However, this stratification has demonstrated low accuracy (62.1%) for diagnosing choledocholithiasis and overly relies on invasive tests, such as endoscopic retrograde cholangiopancreatography (ERCP).^(3,4)^

Endoscopic retrograde cholangiopancreatography is considered the most accurate diagnostic, as well as therapeutic, method for choledocholithiasis.^(3)^ This procedure is often used in patients with a high or moderate risk of choledocholithiasis.^(3)^ However, it is an invasive test that requires use of anesthesia and iodine contrast and has a significant risk of complications (6–15%), which highlights that ERCP should be used with caution after adequate patient selection.^(5,6)^

Recently, magnetic resonance cholangiopancreatography (MRCP) has gained attention as a good alternative for preoperative evaluation of choledocholithiasis.^(7)^ Magnetic resonance cholangiopancreatography is a non-invasive method that has an accuracy similar to that of ERCP: a sensitivity of 91-100% a specificity of 95-100%, and a high negative predictive value. However, it is expensive and not universally available; consequently, its routine use is limited.^(5,7-9)^ Use and availability of magnetic resonance imaging (MRI) are restricted, and even, nonexistent in many locations.^(10)^ Most countries of the world, including Brazil, are undeveloped where healthcare-related resources are scarce.^(11)^ Considering that approximately 15% of patients with cholelithiasis develop asymptomatic choledocholithiasis,^(12,13)^ the use of healthcare resources, whether laboratory or imaging tests, should be based on strict criteria to save unnecessary expenses.

The average cost of an MRCP in the Brazilian public healthcare system is equivalent to US $49.55, according to SUS/CISAMUSEP (*https://cisamusep.org.br/wp-content/uploads/2021/02/tabela_sus_referencia_set-2020.pdf*) registers of 2021. Meanwhile the costs of serum laboratory exams, such as those for total bilirubin, alkaline phosphatase, gamma-glutamyl transferase, and aspartate and alanine transferase, are all less than US $0.65 each.

According to DataSUS (https://datasus.saude.gov.br/), a database referring to the Public Health System in Brazil, the *per capita* expenses regarding complex imaging tests, such as MRCP, have almost doubled in the past 15 years. Additionally, the public health insurance system constantly has high claims due to a lack of income to meet all healthcare demands. Consequently, the medical community must look for alternative methods for choledocolithiasis diagnosis.

## OBJECTIVE

To assess the predictive value of preoperative serum laboratory test results for identifying asymptomatic choledocholithiasis and, in this way, reduce magnetic resonance cholangiopancreatography-related costs.

## METHODS

A retrospective analysis of data of patients aged 21-69 years who underwent preoperative MRCP between June 2018 and May 2019 at our institute was performed. Patients with a history of fluctuating jaundice or biliary pancreatitis, dilatation of the bile ducts on ultrasound, and elevation of canalicular enzymes (AP >100 and GGT >50) underwent MRCP (GE brand, model Optima 360, body of 70cm, 1.5 tesla magnetic field). Demographic and laboratory serum data, including levels of canalicular enzymes, transaminases, and bilirubin, were collected. The interval between serum tests and MRI was 1 month. Data were extracted from procedures performed at *Hospital Municipal da Vila Santa Catarina Dr. Gilson de Cássia Marques de Carvalho; Hospital Israelita Albert Einstein,* São Paulo, Brazil.

The cost of the MRCP is R$ 268.75 *Sistema de Gerenciamento da Tabela de Procedimentos, Medicamentos* and *Órteses, próteses e materiais* of *Sistema Único de Saúde* and that the US Dollar compared to Real in May 2019 was equivalent to R$ 3.8961.^(14)^ Patients with clinical jaundice, direct bilirubin level >0.8mg/dL, pancreatitis, associated weight loss, or choledocholithiasis confirmed by ultrasonography were excluded from analyses.

In this case-control study, cases of choledocholithiasis confirmed by MRCP were compared with cases without choledocholithiasis and the diagnostic capacities of the preoperative serum laboratory test results were analyzed. Binary, univariate, and multivariate logistic regression analyses were performed. The diagnostic capacities of the examination results were evaluated using receiver operating characteristic (ROC) curves. The maximum Youden index values were used as the cut-off points for analyzing the diagnostic performance measures. The data were analyzed using STATA (version 16.1). A p value ≤0.05 was considered statistically significant.

The project was approved by the Research Ethics Committee of *Hospital Israelita Albert Einstein,* CAAE: 24400719.6.0000.0071; #4.057.325, which exempted the need for informed consent.

## RESULTS

A total of 104 patients were included, with a median age of 52 years (21-69), and 70.2% were women. Eight patients were diagnosed with asymptomatic choledocholithiasis with MRCP. The baseline characteristics of the patients are shown in table 1.

**Table 1:** Baseline characteristics of the patients

	Total	With choledocolithiasis	Without choledocolithiasis
n	104	8	96
Gender (Male:Female)	31:73	2:6	29:67
Age (years) (Min-Max)	52 (21-69)	38 (23-61)	52 (21-69)
BMI (kg/m^2^) (Min-Max)	30 (19-46)	29 (20-41)	30 (19-46)
Total bilirubin	0.41 (0.1-2.9)	0.4 (0.21-0.9)	0.44 (0.1-2.9)
Direct bilirubin	0.2 (0.09-0.8)	0.11 (0.1-0.4)	0.2 (0.09-0.8)
Alkaline phosphatase	82 (47-236)	107 (78-236)	80 (47-229)
Gamma-glutamyl transferase	98 (8-987)	104 (41-394)	98 (8-987)
Aspartate aminotransferase	26 (12-213)	22 (15-56)	27 (12-213)
Alanine aminotransferase	38 (10-207)	38 (13-121)	39 (10-207)

BMI: body mass index.

**Figure 1:**
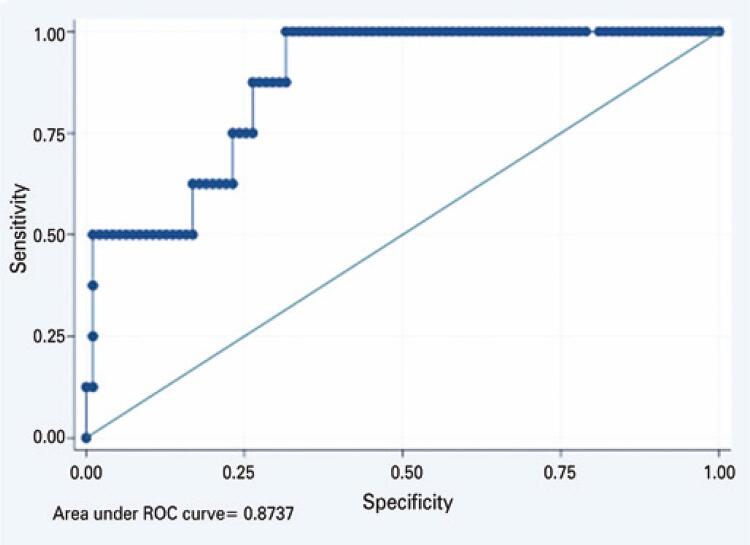
The ROC curve for the final model with the variables age and alkaline phosphatase, for the outcome choledocholithiasis

In the binary logistic regression analysis, age (odds ratio [OR]: 0.92; 95% confidence interval [CI]: 0.86-0.98; p=0.016) and serum ALP levels (OR= 1.02; 95%CI: 1.01-1.05; p=0.004) were selected for the final model. Therefore, the final model was constructed as: logit(p) = -1.684736 + (-0.0841961 x “Age”) + (0.02761 x “Alkaline Phosphatase”). Figure 1 represents the ROC curve of the final model, with the selected variables “Age” and “Alkaline Phosphatase.” Table 2 presents the results of the univariate and multivariate analyses.

**Table 2:** Univariate and multivariate analyses for the outcome choledocholithiasis diagnosed by magnetic resonance cholangiopancreatography

Independent variables	Univariate analysis			95%CI		Multivariate analysis			95%CI	
			
	OR	SE	P>|z|	Inferior	Superior	OR	SE	P>|z|	Inferior	Superior
Age	0.936	0.028	0.026^†^	0.882	0.992	0.919	0.032	0.016^†^	0.859	0.984
Female	1.298	1.099	0.758	0.247	6.819					
BMI (kg/m^2^)	0.978	0.054	0.687	0.877	1.09					
Total bilirubin	0.374	0.487	0.45	0.029	4.786					
Direct bilirubin	0.039	0.169	0.456	7.39	2.021					
Alkaline phosphatase	1.024	0.008	0.004^†^	1.007	1.04	1.028	0.01	0.004^†^	1.009	1.047
Gamma-glutamyl transferase	1.001	0.002	0.629	0.997	1.005					
Aspartate aminotransferase	0.989	0.021	0.593	0.948	1.031					
Alanine aminotransferase	1.002	0.011	0.865	0.981	1.023					

^†^ Standard error. p<0.05.

OR: odds ratio; 95%CI: 95% confidence interval; BMI: body mass index; SE: standard error.

Thus, among the serum laboratory test results, only ALP level was significantly capable of predicting choledocholithiasis. The maximum value of the Youden index for ALP level was 78U/L. For this cut-off point, the sensitivity of the serum ALP test was 97.6% (95%CI: 87.4-99.9), and the specificity was 72.6% (95%CI: 59.8-83.1). Table 3 presents the diagnostic performance measures of serum ALP levels above the 78U/L cut-off point.

**Table 3:** Serum alkaline phosphatase diagnostic performance at or above the 78U/L cut-off point, determined by the highest Youden index value

		95%CI
Sensitivity, %	97.62	87.43-99.94
Specificity, %	72.58	59.77-83.15
Positive likelihood ratio	3.56	2.37-5.35
Negative likelihood ratio	0.03	0.00-0.23
Positive predictive value, %	70.69	61.60-78.38
Negative predictive value, %	97.83	86.58-99.68
Accuracy, %	82.69	74.03-89.41

95%CI: confidence interval.

In our study, if only patients who had ALP values ≥78U/L patients had undergone MR cholangiography, no patient with choledocholithiasis would have been misdiagnosed before surgery. Of the 41 patients with ALP <78U/L who did not undergo MRI, none had choledocholithiasis. Of the 55 patients with ALP ≥78U/L, 8 had choledocholithiasis. In this hypothetical situation, the average expense of MR would have decreased from R$ 268.75 to R$ 142.13 for each patient in this series (a 47.1% reduction in cost).

## DISCUSSION

Serum ALP levels can help predict asymptomatic choledocholithiasis and negate the requirement of MRI in several patients, especially when supported by other clinical and imaging findings.

The impact of cholangioresonance-related costs on the Brazilian public healthcare system is high. Meanwhile, the costs of serum laboratory examinations, such as those for total bilirubin, ALP, gamma-glutamyl transferase, aspartate, and alanine transferase, are all less than US$ 1. Herein, we attempted to find an efficient way to mitigate the expense of cholangioresonance, focusing on the aforementioned serum laboratory examinations, since the total cost of cholangioresonance has practically doubled in the last 15 years. In the hypothetical situation brought about by the observed results, the average expense of MR cholangiography would have decreased by almost half for each patient included in the study (47.1% reduction in cholangioresonance-related costs).

According to World Population Prospects (2019 data), the combined population of underdeveloped and developing countries corresponds to 67.9% of the world population;^(15)^ additionally, developing countries will account for 80% of the world population in the next 10 years. Healthcare management in these countries are restricted due to low income and a highly heterogeneous distribution of resources. In this context, strategies aimed at reducing the costs of healthcare services may have a great impact, as they make it possible to expand access to healthcare services for the general population. The use of ALP as a predictor for choledocholithiasis in clinically-suspected patients would reduce cholangioresonance-related costs without compromising the quality of care provided.

Previous studies have demonstrated the nuances related to the role of each biochemical marker in choledocholithiasis.^(16-18)^ Most previous studies have demonstrated greater accuracy of gamma-glutamyl transferase than of ALP for diagnosing choledocholithiasis.^(16-18)^ However, for the most part, these studies did not consider different intervals and their respective sensitivities and specificities.^(16-18)^ In contrast, the present study, in addition to considering distinct gaps, highlighted ALP as a significant predictor with a cut-off value of 78U/L and sensitivity and specificity of 97.6% and 72.6%, respectively.

In our model, in addition to ALP level, age was an independent variable associated with a higher risk of choledocholithiasis. This finding is consistent with the pathophysiology of gallstones. There is a higher incidence of factors that favor biliary stasis and the precipitation of gallstones around the fourth decade of life.^(19)^

In addition, a study shows that gamma-glutamyl transferase is more sensitive for diagnosing choledocholithiasis.^(19)^ In our study, gamma-glutamyl transferase was not a significant factor, probably because of the small number of cases studied.

Most current guidelines indicate the use of cholangioresonance for patients with an intermediate risk of choledocholithiasis (patients with a diagnostic probability of 10%–50%).^(20)^ Risk determination takes into account the presence of jaundice, serum bilirubin levels, abnormal liver biochemistry tests, as well as the presence of bile duct dilatation; some studies also consider age >55 years as an additional factor.^(18)^ Although studies have considered the levels of canalicular enzymes to determine the risk of choledocholithiasis, the relevance of each marker and their respective cut-off values are not well established.^(18,20)^ Many authors consider that patients at high risk for choledocholithiasis should undergo ERCP, and low-risk patients should undergo cholecystectomy with or without intraoperative cholangiography.^(19,20)^ Intraoperative laparoscopic ultrasonography can also be used in this context; however, its applicability is limited.^(20)^ To our knowledge, no previous study has demonstrated the effectiveness of serum markers for diagnosing choledocholithiasis to mitigate the need for expensive non-invasive imaging tests such as cholangioresonance.

Despite the relevant results, this study has limitations. The study was retrospective in nature and performed at a single center with a limited number of patients. Furthermore, other differential diagnoses for obstructive jaundice should be considered. Differential diagnoses include hepatobiliopancreatic neoplasms, for which imaging tests, such as MRI, are indispensable. Therefore, laboratory test results should always be interpreted based on a detailed clinical evaluation of the patient, and imaging examinations should only be performed according to the clinical context.

## CONCLUSION

Serum alkaline phosphatase levels may aid preoperative diagnosis of asymptomatic choledocholithiasis. After a global clinical evaluation of the patient, the serum laboratory findings may contribute to a reduction in magnetic resonance cholangiography-related healthcare costs.
